# Biallelic loss-of-function mutation in *NIK* causes a primary immunodeficiency with multifaceted aberrant lymphoid immunity

**DOI:** 10.1038/ncomms6360

**Published:** 2014-11-19

**Authors:** Katharina L. Willmann, Stefanie Klaver, Figen Doğu, Elisangela Santos-Valente, Wojciech Garncarz, Ivan Bilic, Emily Mace, Elisabeth Salzer, Cecilia Domínguez Conde, Heiko Sic, Peter Májek, Pinaki P. Banerjee, Gregory I. Vladimer, Şule Haskoloğlu, Musa Gökalp Bolkent, Alphan Küpesiz, Antonio Condino-Neto, Jacques Colinge, Giulio Superti-Furga, Winfried F. Pickl, Menno C. van Zelm, Hermann Eibel, Jordan S. Orange, Aydan Ikincioğulları, Kaan Boztuğ

**Affiliations:** 1 CeMM Research Center for Molecular Medicine of the Austrian Academy of Sciences, Vienna 1090, Austria; 2 Department of Immunology, Institute of Biomedical Sciences, University of São Paulo, São Paulo 05508-900, Brazil; 3 Department of Pediatric Immunology and Allergy, Ankara University Medical School, Ankara 06100, Turkey; 4 Center for Human Immunobiology, Baylor College of Medicine and Texas Children’s Hospital, Houston, Texas 77030, USA; 5 Centre of Chronic Immunodeficiency, University Medical Centre Freiburg, Freiburg 79180, Germany; 6 Department of Pediatric Hematology, Akdeniz University Medical School, Antalya 07985, Turkey; 7 Christian Doppler Laboratory for Immunomodulation and Institute of Immunology, Center for Pathophysiology, Infectiology and Immunology, Medical University of Vienna, Vienna 1090, Austria; 8 Department of Immunology, Erasmus MC, University Medical Center, Rotterdam 3015GE, The Netherlands; 9 Department of Paediatrics and Adolescent Medicine, Medical University of Vienna, Vienna 1090, Austria

## Abstract

Primary immunodeficiency disorders enable identification of genes with crucial roles in the human immune system. Here we study patients suffering from recurrent bacterial, viral and *Cryptosporidium* infections, and identify a biallelic mutation in the *MAP3K14* gene encoding NIK (NF-κB-inducing kinase). Loss of kinase activity of mutant NIK, predicted by *in silico* analysis and confirmed by functional assays, leads to defective activation of both canonical and non-canonical NF-κB signalling. Patients with mutated *NIK* exhibit B-cell lymphopenia, decreased frequencies of class-switched memory B cells and hypogammaglobulinemia due to impaired B-cell survival, and impaired ICOSL expression. Although overall T-cell numbers are normal, both follicular helper and memory T cells are perturbed. Natural killer (NK) cells are decreased and exhibit defective activation, leading to impaired formation of NK-cell immunological synapses. Collectively, our data illustrate the non-redundant role for NIK in human immune responses, demonstrating that loss-of-function mutations in *NIK* can cause multiple aberrations of lymphoid immunity.

## Abstract (web summary)

Primary immunodeficiency disorders can be used to identify key immune functions. Here, the authors identify a biallelic mutation in the gene encoding NF-κB-inducing kinase in a family suffering a range of infections, and show that it causes defects in NK and T-cell function and has broad effects on B-cell function.

## Graphical abstract

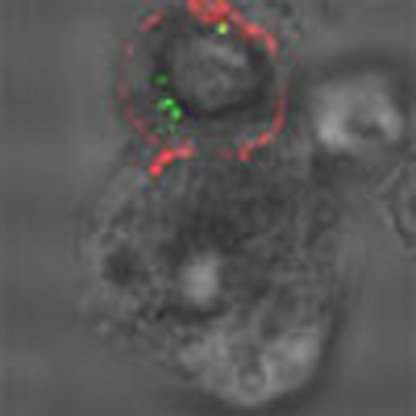

Primary immunodeficiency disorders represent unique models to identify factors essential for host defense and immune homeostasis. In humans, development of mature B cells from immature precursor cells is critically dependent on signalling pathways downstream of B-cell receptor (BCR) and on tumour necrosis factor-α (TNFα) receptor superfamily members including BAFF receptor (BAFFR), TACI and CD40 (reviewed in ref. 1). BAFFR signals are needed to mature beyond the transitional B-cell stage^2^, while lymphotoxin-α1/β2 (LTβ) and CD40 ligand (CD40L) are required for thymic and secondary lymphoid organ structure, respectively^3^. CD40-mediated signalling additionally orchestrates processes dependent on CD4^+^ T-helper cells such as class-switch recombination (CSR) and somatic hypermutation (SHM) in the germinal centre (GC) reaction and CD8^+^ cytotoxic T-cell memory^4^.

BAFFR, CD40 and LTβ receptors transmit signals through the non-canonical nuclear factor-κB (NF-κB) pathway (reviewed in ref. 5), which induces proteolytic processing of p100 to p52 (ref. 6). Together with RelB, p52 forms a heterodimer that upon nuclear translocation functions as transcriptional activator of a subset of NF-κB target genes^5^. Processing of p100 depends on the phosphorylation of the serine residues 866 and 870, which is controlled by the MAP3 kinase–kinase–kinase NIK (NF-κB inducing kinase, MAP3K14)^6^ through NIK’s substrate IκB kinase α (IKKα)^7^. Non-canonical NF-κB signalling is controlled by TNF receptor associated factor (TRAF) proteins TRAF2 and NIK’s negative regulator TRAF3, whereby a TRAF3-containing complex continuously targets NIK for degradation under steady-state conditions^5^. On receptor activation, TRAF3 is degraded and NIK protein levels can accumulate, allowing NIK to phosphorylate and activate downstream effectors.

To date, human patients carrying mutations in *MAP3K14* have not yet been described. In *Nik* mutant mice (*aly*; alymphoplasia)^8^,^9^ and knockout animals^3^, lymph nodes, Peyer’s patches as well as splenic and thymic structures are severely disorganized. In addition, B-cell numbers are reduced and immunoglobulin (Ig) serum levels are decreased leading to humoral immunodeficiency. Although the function of NIK in B lymphocytes has been well established, the role of NIK-dependent signalling for T and natural killer (NK) lymphocytes is less well understood.

Here we report a combined immunodeficiency syndrome caused by biallelic mutations in the gene encoding NIK, encompassing B-cell lymphopenia and impaired memory B-cell differentiation. We also identify abnormal NK-cell development and function, as well as aberrant T-cell responses, indicating that biallelic loss-of-function mutations in *NIK* cause a hitherto unrecognized, pervasive combined immunodeficiency syndrome.

## Results

### Identification of a homozygous mutation in *MAP3K14*

We studied a large consanguineous pedigree with two patients (termed P1 and P2) who showed signs of combined immunodeficiency including recurrent, severe bacterial and viral infections and *Cryptosporidium* infection (Supplementary Fig. 1a,b and Supplementary Tables 1 and 2; see Supplementary Note for further clinical course details). Investigation for known genetic aetiologies of defective CSR including CD40 and CD40L deficiencies and gain-of-function *PIK3CD* mutations^10^,^11^ was performed; however, no mutation was identified.

**Figure 1:**
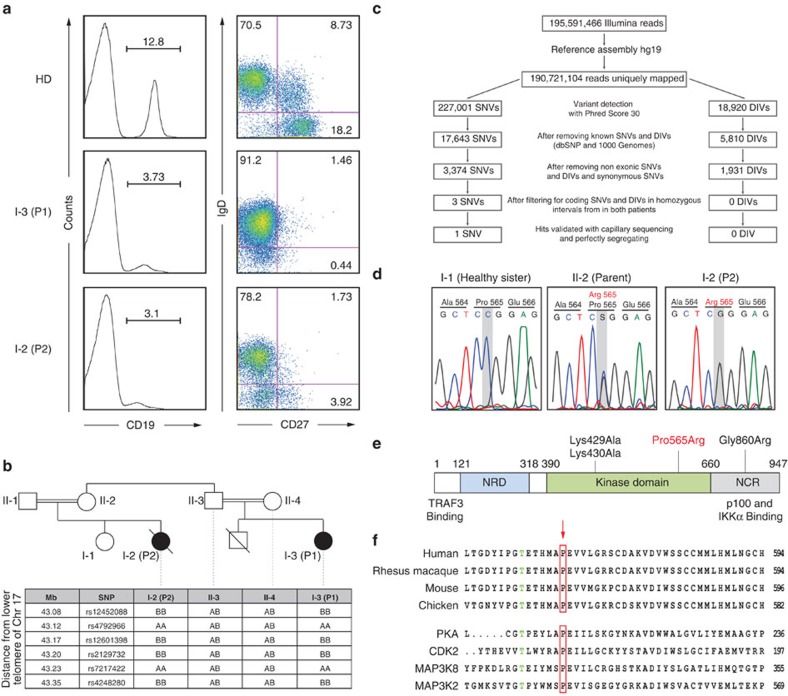
Identification of *MAP3K14/NIK* mutation in patients with defective B cells. (**a**) Flow cytometry plots illustrating decreased CD19^+^ B cells and decreased CD27^+^IgD^−^ class-switched memory B cells in P1 and P2. Plots representative of three independent experiments. (**b**) SNP array based homozygosity mapping revealed several homozygous candidate intervals shared between both patients, including an interval on chromosome 17q21, described in the box. (**c**) Scheme of exome sequencing workflow and filtering strategy. SNVs, single nucleotide variants; DIVs, deletions and insertions variants. (**d**) Capillary DNA sequencing of the regions adjacent to the nonsense mutation in *MAP3K14* in P2 and core family members. Chromatograms shown for a healthy sister of P2, the mother of P2 and P2. The mutated residue is indicated by a grey box. (**e**) Schematic representation of the NIK protein domain structure. NRD, negative regulatory domain (blue); kinase domain (green); NCR, non-catalytic region (grey)^79^. Red label indicates the amino acid change in P1 and P2. Black labels indicate the catalytic inactive mutant NIK^Lys429Ala/Lys430Ala^ and the murine *aly/aly* mutant (Gly860Arg). (**f**) Amino acid sequence conservation of the region adjacent to Pro565 across species as well as a panel of human serine/threonine kinases. Red arrow indicates Pro565 mutated in P1 and P2; Thr559 printed in green.

Immunological assessment in both affected patients revealed decreased immunoglobulin levels (Supplementary Table 1) and decreased numbers of both B and NK cells, while T-cell numbers were within normal age-adjusted ranges (Supplementary Table 3). As decreased immunoglobulin levels and B-cell numbers suggested impaired B-cell development and function, we performed flow cytometry-based immunophenotyping to assess the relative frequencies of CD27^+^ memory B-cell populations. Both patients showed a relative reduction of total CD19^+^ B cells in peripheral blood (Fig. 1a). Absolute blood cell counts revealed B lymphopenia in P2, while B-cell numbers in P1 were in the age-matched lower normal range (Supplementary Table 3). Patients had decreased CD19^+^CD27^+^IgD^+^ marginal zone-like/innate B cells and CD19^+^CD27^+^IgD^−^ class-switched memory B cells compared with controls^12^, suggesting defects in late stages of B-cell development and activation (Fig. 1a).

Given the consanguineous background, an autosomal-recessive inheritance mode was assumed. To unveil the presumed monogenetic cause of disease, single-nucleotide polymorphism (SNP) array-based homozygosity mapping of P1 and P2 (Fig. 1b and Supplementary Table 4) was combined with exome sequencing (ES) (Fig. 1c) of P1. Single-nucleotide variants (SNVs) and insertion/deletion variants resulting from ES were filtered for those present inside homozygous candidate intervals shared between both affected patients. Synonymous and non-coding variants were excluded. We identified a single homozygous variant on chromosome 17q21 in *MAP3K14* (c. C1694G, p. Pro565Arg) present in both patients (Fig. 1d and Supplementary Fig. 1), which was not detected in dbSNPbuild137, 1000Genomes, ENSEMBL, UCSC, NCBI or EVS (Exome Variant Server) public SNP databases. The Pro565 residue of NIK is located within the kinase domain of the protein (Fig. 1e) and is highly conserved throughout evolution (Fig. 1f). The exchange from proline to arginine at this position was predicted as highly deleterious using the functional prediction algorithms Polyphen-2 and SIFT with maximum scores (1.0 and 0.0, respectively).

### Effects of NIK^Pro565Arg^ on kinase function

**Figure 2:**
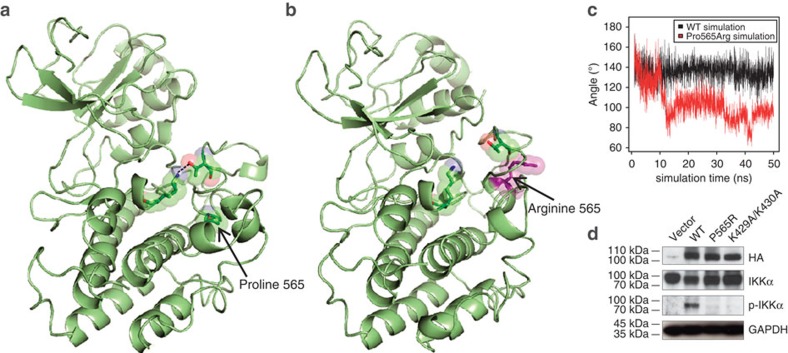
NIK^Pro565Arg^ is structurally altered and catalytically impaired. (**a**) Structure prediction of NIK^wild-type^ catalytic domain from coarse-grained molecular dynamics simulation. Pro565, Thr559 and Lys517 residues are displayed as sticks and transparent molecular spheres. Dashed line represents hydrogen bond between Lys517 and Thr559. (**b**) Structure prediction of NIK^Pro565Arg^ from coarse-grained molecular dynamics simulation. Arg565 residue is displayed as sticks and transparent molecular spheres (violet). (**c**) Molecular dynamics simulation of wild-type NIK and NIK^Pro565Arg^. Profile of the angle of Thr559-CA, Thr559-CM and Lys517-CM. The angle of Thr559-CA, Thr559-CM and Lys517-CM constantly approaches 150° (close to the value observed in the NIK^wild-type^ experimental X-ray structure), while it rapidly transitions to 90° (hydrogen bonding unfavourable) in the simulation of NIK^Pro565Arg^. CA, alpha carbon; CM, centre of side chain mass. (**d**) Analysis of kinase activity of NIK variants expressed in HEK293 cells. HA-tagged NIK variants NIK^wild-type^, NIK^Pro565Arg^ and the catalytically inactive mutant NIK^Lys429Ala/Lys430Ala^ were transfected into HEK293 cells and levels of total and phosphorylated IKKα were detected by immunoblot. Anti-HA and anti-GAPDH blots were used as loading controls for NIK and total protein, respectively. Blots are representative of three independent experiments. All uncropped blots can be seen in Supplementary Fig. 9.

Pro565 forms part of the APE motif within a helix in the activation segment of the kinase^13^. This motif is conserved in NIK from various vertebrate phyla as well as in orthologous serine/threonine kinases (Fig. 1f). An exchange of a non-polar, conformationally rigid amino acid as in NIK^Pro565Arg^ may have an impact on protein folding and function. The protein stability analysis tool CUPSAT predicted that the overall stability of NIK^Pro565Arg^ may be compromised (Supplementary Table 5). Coarse-grained molecular dynamics simulation of NIK^wild-type^ and NIK^Pro565Arg^ showed conformational changes within the kinase domain. Notably, the nearby Thr559 residue has been reported to form a hydrogen bond with Lys517 in the catalytic loop (Fig. 2a) and mutation of Thr559 has been found to reduce kinase activity^14^,^15^. In the NIK^wild-type^ simulation, Pro565 remains buried within the protein in the vicinity of the ATP-coordinating centre, allowing hydrogen bond formation between Lys517 and Thr559 (Fig. 2a). In the NIK^Pro565Arg^ simulation, the mutated arginine residue transitions towards the protein surface, thereby increasingly contacting the surrounding solvent and repositioning adjacent helices. In the simulation, Arg565 prevents Thr559 from forming a hydrogen bond with Lys517 in the ATP-coordinating centre, thereby impairing the kinase activity of NIK (Fig. 2b,c).

To experimentally assess the effect of the mutation, we analysed the kinase activity of NIK^Pro565Arg^ compared with NIK^wild-type^ and the catalytically inactive mutant NIK^Lys429Ala/Lys430Ala^ (ref. 16) by testing NIK-dependent phosphorylation of IKKα. Recombinantly expressed NIK^wild-type^, but not NIK^Pro565Arg^ or NIK^Lys429Ala/Lys430Ala^, could phosphorylate both endogenous (Fig. 2d) and co-expressed IKKα (Supplementary Fig. 2) in HEK293 cells. These data demonstrate that NIK^Pro565Arg^ represents a loss-of-function mutation with respect to abolished kinase activity towards its direct target IKKα.

### Defective non-canonical and canonical NF-κB signalling

**Figure 3:**
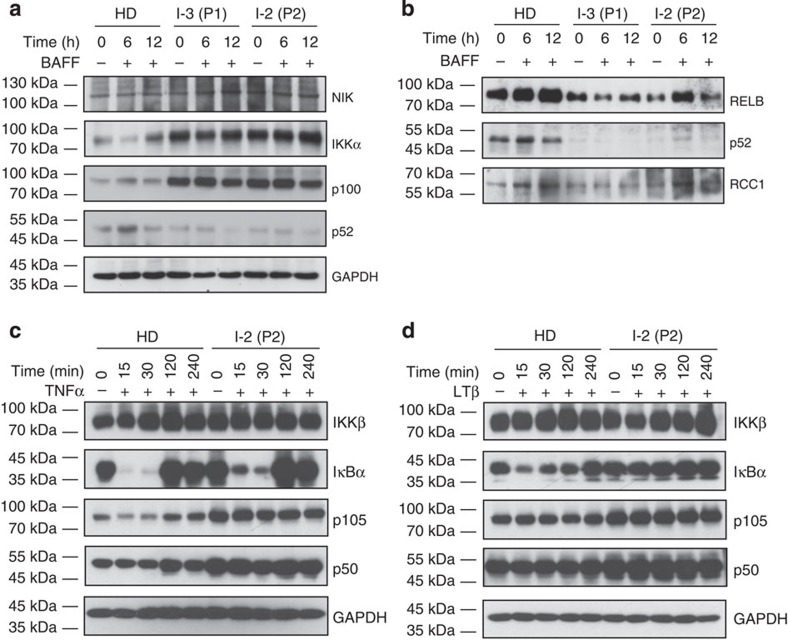
Defective canonical and non-canonical NF-κB pathway function. (**a**) Immunoblot analysis of whole cell lysates of B-LCL stimulated with hBAFF. Healthy donor, P1 and P2 derived B-LCL were used. Blots were probed for non-canonical NF-κB pathway components NIK, IKKα, p100/p52 and sample processing control GAPDH. (**b**) Immunoblot analysis of nuclear extracts of B-LCL stimulated with hBAFF in healthy donor, P1- and P2-derived B-LCL. RCC1 was used as nuclear sample processing control. (**a**,**b**) Blots are representative of two independent experiments. All uncropped blots can be seen in Supplementary Fig. 9. (**c**) Immunoblot analysis of whole cell lysates of healthy donor- and P2-derived fibroblasts after TNFα stimulation. Blots were probed for canonical NF-κB pathway components IKKβ, IκBα, p105/p50 and GAPDH as loading control. (**d**) Immunoblot analysis of whole cell lysates of healthy donor- and P2-derived fibroblasts after LTβ stimulation. Blots were probed for canonical NF-κB pathway components IKKβ, IκBα, p105/p50 and loading control GAPDH. (**c**,**d**) Blots represent one experiment. All uncropped blots can be seen in Supplementary Fig. 9.

Processing of p100 into p52 and nuclear translocation of the p52/RelB complex is an essential step following NIK activation^6^. Therefore, we studied activity of the non-canonical NF-κB pathway in response to activation with BAFF and LTβ, respectively. In patient-derived Epstein–Barr virus-immortalized lymphoblastoid cell lines (B-LCL), total NIK protein levels (which are tightly controlled via proteolysis^5^) were unaffected (Fig. 3a). However, levels of its immediate downstream target IKKα were elevated and p100 accumulated already before BAFF-mediated BAFFR ligation (Fig. 3a), possibly reflecting pre-activation of the NF-κB pathway in B-LCL by viral proteins^17^. Despite p100 accumulation, p52 protein levels were decreased (Fig. 3a), resulting in a severely reduced nuclear content of p52 and a lower nuclear content of RelB in patient-derived cells (Fig. 3b), demonstrating functional insufficiency of the non-canonical NF-κB pathway in patient-derived cells.

**Figure 4:**
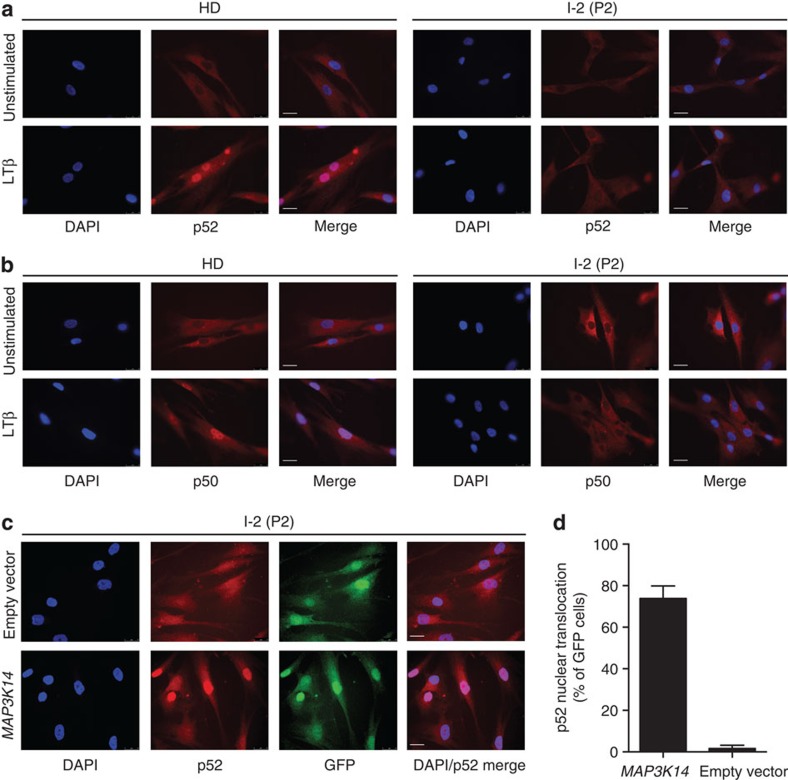
Defective nuclear translocation of canonical and non-canonical NF-κB and rescue by ectopic expression of NIK^wild-type^. (**a**,**b**) Immunofluorescence analysis of healthy donor and P2 fibroblasts stimulated with LTβ and stained with DAPI, (**a**) anti-p100/p52 and (**b**) anti-p105/p50 antibodies, respectively. White bar indicates 25 μm. (**c**) Immunofluorescence analysis of P2 fibroblasts after retroviral transduction with *MAP3K14* or empty vector, both coexpressing GFP. Cells were stained with DAPI, anti-GFP and anti-p100/p52 antibody. White bar indicates 25 μm. (**d**) Quantification of reconstitution experiment in P2 fibroblasts shown in **c**. GFP-positive cells (representing cells transduced with *MAP3K14* or empty vector, respectively) were scored for p52 nuclear translocation events, indicated as mean percentage of total (±s.e.m.). 86 (*MAP3K14*) and 97 (empty vector) cells were scored, respectively. (**c**,**d**) Images and quantification are representative of two independent experiments.

Next, we tested the effect of NIK^Pro565Arg^ in NF-κB activation upon LTβ stimulation, which is strictly dependent on NIK^18^. LTβ can activate non-canonical NF-κB signalling as well as the IKKα-IKKβ-NEMO complex mediating nuclear translocation of canonical NF-κB complexes^19^. Stimulation of patient fibroblasts with the NIK-independent canonical NF-κB activator TNFα led to a rapid decay of IκBα, a hallmark event of canonical NF-κB signalling (Fig. 3c). In contrast, patient cells failed to induce IκBα decay after LTβ stimulation (Fig. 3d). Consistent with these findings, immunofluorescent staining showed defective nuclear translocation of both p52 and p50 in patient primary fibroblasts upon LTβ stimulation (Fig. 4a,b).

To demonstrate the causative role of NIK^Pro565Arg^ for deficient non-canonical NF-κB signalling, we performed retroviral-mediated gene transfer of *MAP3K14* into patient fibroblasts. Expression of NIK^wild-type^ reactivated non-canonical NF-κB signalling manifesting in nuclear translocation of p52 (Fig. 4c,d), demonstrating that the presence of functional NIK protein is the limiting factor for p100 activation.

Although NIK may have IKKα-independent functions^20^, the main function of the protein comprises the catalytic activity as a kinase and activation of the signalling cascade leading to NF-κB translocation. As the NIK^Pro565Arg^ mutant is catalytically inactive and therefore deleterious to these functions, the phenotype caused by NIK^Pro565Arg^ is hereafter also referred to as functional NIK deficiency.

### Reduced survival of mature B cells

The spontaneous *Map3k14* mouse *aly* mutant^8^,^9^ and *Map3k14* knockout mice^3^ show reduced mature B-cell numbers and decreased Ig serum levels, resulting in defects in both antibody and cellular immune responses. In addition, non-canonical NF-κB signalling mediated by Nik controls CSR, in particular to IgA isotype^21^. Accordingly, patients bearing NIK^Pro565Arg^ had severely reduced total B-cell counts and impaired generation of CD27^+^IgD^−^ class-switched memory B cells in the peripheral blood (Fig. 1a), leading to chronically reduced IgA titres in both patients and to reduced IgG levels in P1 at the age of 10 months (Supplementary Table 1) prompting intravenous immunoglobulin substitution. This led us to test whether NIK is involved in CSR and SHM, processes essential for the generation of high-affinity antibodies.

**Figure 5:**
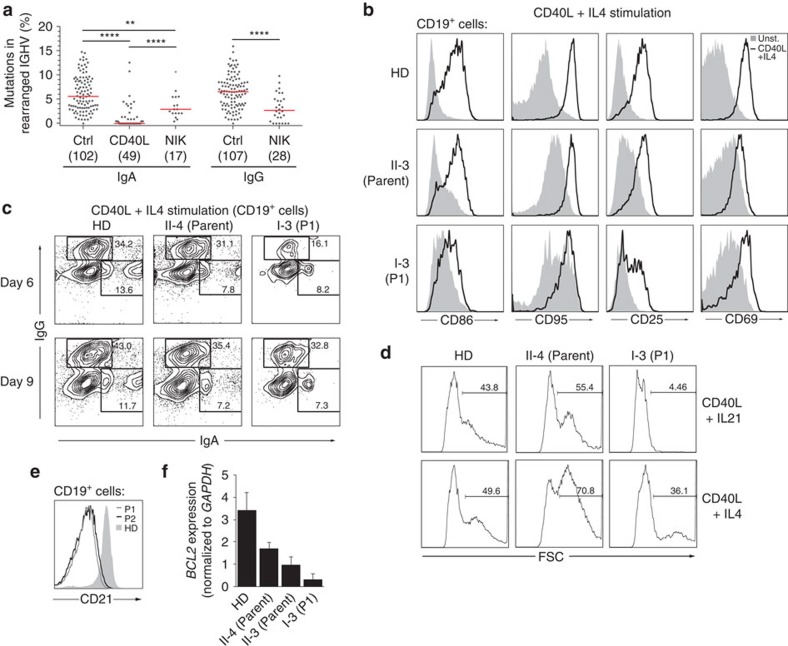
Aberrant B-cell phenotype. (**a**) Analysis of mutation frequency in percent mutated bases in rearranged variable regions of switched Ig heavy chains (*IGHV*) of IgG and IgA isotypes in healthy donors and patients. Analysed sequences from both patients are represented combined. The number of sequences analysed is indicated in brackets. (**b**) Flow cytometric analysis of the expression of activation markers CD86, CD95, CD25 and CD69, 1 day after *in vitro* stimulation with CD40L and IL4. Plots are representative of three independent experiments. (**c**) Flow cytometry analysis of the expression of class-switch markers IgG and IgA at day 6 and 9 after *in vitro* stimulation with CD40L and IL4. (**d**) *In vitro* proliferation of primary lymphocytes after stimulation with CD40L and IL21 or CD40L and IL4. The forward scatter gate indicates percentages of proliferating blasts in parent or patient P1 cultures after 9 days. (**e**) Flow cytometry analysis of the CD21 expression on CD19^+^ B cells of a healthy donor (filled grey), P1 (grey line) and P2 (black line). (**c**–**e**) Plots represent one experiment. (**f**) Quantitative reverse transcriptase–PCR analysis of *BCL2* expression in sorted peripheral blood naive B cells. *BCL2* transcript expression was normalized to *GAPDH* expression. Mean fold enrichment from one experiment is shown. Error bars denote ±s.d. from three technical replicates.

To study the occurrence of SHM in B cells, we analysed the mutation frequency in rearranged variable regions of the Ig heavy chain (*IGHV*) genes by cloning and sequencing the *IGHV3* and *IGHV4* rearranged gene families of both γ- and α-chain immunoglobulin transcripts (Cγ and Cα). The percentage of mutations within the analysed *IGHV* regions was significantly reduced in NIK^Pro565Arg^-bearing patients compared with age-matched healthy donors, although not as severely as in CD40L-deficient patients who showed near-complete absence of mutations in *IGHV* Cα and completely lacked Cγ transcripts (Fig. 5a)^22^.

Next, we investigated activation and CSR capacity of NIK^Pro565Arg^ B cells by stimulating peripheral blood mononuclear cells (PBMCs) with a range of stimuli. Patient B cells were able to respond to stimulation with CD40L and IL4 by upregulating the activation markers CD95 and CD69, as well as the costimulatory molecule CD86, although to a lesser extent than B cells from a healthy donor (Fig. 5b). Notably, patient B cells were only partially able to upregulate the activation marker CD25, suggesting impaired IL2-mediated survival and proliferation^23^. As NIK^Pro565Arg^ B cells were largely able to upregulate the aforementioned activation markers, we further tested their proliferation capacity and ability to undergo CSR in response to CD40L and IL4 stimulation. Indeed, we observed a progressive increase in the percentage of DAPI (4',6-diamidino-2-phenylindole)-negative blasts in NIK^Pro565Arg^ mutant cells (Supplementary Fig. 3a) over a course of 9 days, consistent with activation and proliferation of cells upon stimulation with CD40L and IL4 (ref. 24). Furthermore, B cells with mutated NIK underwent CSR to IgG *in vitro*, albeit with reduced frequency at day 6 when compared with controls (Fig. 5c), probably due to delayed lymphocyte proliferation (Supplementary Fig. 3a). Concomitant to proliferative outgrowth of lymphocytes at day 9, CSR to IgG was restored to levels comparable to control cells (Fig. 5c). NIK^Pro565Arg^ mutant B cells did not proliferate after *in vitro* stimulation with CD40L/IL21 (Fig. 5d and Supplementary Fig. 3b), despite intact expression of IL21 receptor on patient B cells (Supplementary Fig. 4a) nor after *in vitro* stimulation with anti-IgM/CpG or anti-IgM/CpG/BAFF-Fc (Supplementary Fig. 3c,d). These data demonstrated the inability of NIK^Pro565Arg^ B cells to respond to BCR, TLR9, IL21R and/or BAFFR stimulation. Only CD40L/IL4 stimulation, known to mediate survival and proliferation of primary B cells^25^,^26^, could induce cell proliferation (Fig. 5d and Supplementary Fig. 3a), suggesting that intact NIK is required to relay signals essential for survival and proliferation of activated mature B-cell populations.

Given the partial phenotypic overlap of functional NIK deficiency and IL21 (receptor) deficiency^27^,^28^ (for example, colitis, susceptibility to *Cryptosporidium* infection, hypogammaglobulinemia and decreased frequencies of class-switched B cells, defective antigen-specific T-cell proliferation and impaired NK-cell cytotoxicity), we sought to exclude an involvement of NIK in IL21-mediated signalling in B cells. Indeed, although classical signalling via STAT3 (signal transducer and activator of transcription 3) phosphorylation was readily observed after stimulating sorted B cells with IL21, no activation of p100 processing could be detected (Supplementary Fig. 5).

As NIK is an integral component of the non-canonical NF-κB pathway downstream of the BAFFR, which plays a key role in mature B-cell survival^29^, we investigated whether functional NIK deficiency resembles phenotypes found in BAFFR deficiency^2^,^30^. Similar to findings in *Baffr*^*−/−*^ mice^30^, B cells of both patients showed lower cell surface expression of CD21, involved in pro-survival signalling on B cells^31^ (Fig. 5e). This observation prompted us to investigate whether the NIK^Pro565Arg^ mutant affects the expression of anti-apoptotic genes *BCL2*, *BCL2L1* and *MCL1* by quantitative reverse transcriptase–PCR in sorted naïve mature CD19^+^CD27^−^IgD^+^ B cells. From the transcripts tested, *BCL2* expression was markedly downregulated in patient naive B cells compared with heterozygous parent and healthy donor controls (Fig. 5f and Supplementary Fig. 4b). To exclude that functional NIK deficiency ablated BAFFR expression, we analysed surface BAFFR levels by flow cytometry on PBMCs stimulated *in vitro* with CD40L and IL4 for 9 days. BAFFR expression on B cells from P1 was comparable to B cells from control parent or healthy donor (Supplementary Fig. 4c).

BAFFR-deficient patients display a partial block in development beyond the transitional CD19^+^CD21^low/intermediate^ B-cell stage^2^. As NIK^Pro565Arg^ patient peripheral B cells had overall a CD19^+^CD21^low/intermediate^ phenotype, we tested the expression of the alternative transitional B-cell markers IgM, CD10, CD38 and CD5. Transitional B cells (defined as CD19^+^IgM^hi^IgD^+^ and CD19^+^CD23^−^CD27^−^CD5^+^IgM^hi^, respectively) were increased, in particular the CD19^+^CD38^+^CD10^−^ transitional T2 population, indicating a partial block in B-cell maturation (Supplementary Fig. 4d,e). Taken together, the NIK^Pro565Arg^ loss-of-function mutant causes a partial block of B-cell development between transitional and naive mature B-cell stages accompanied by impaired survival of mature peripheral B cells.

### Aberrant T-cell phenotype and antigen-specific proliferation

**Figure 6:**
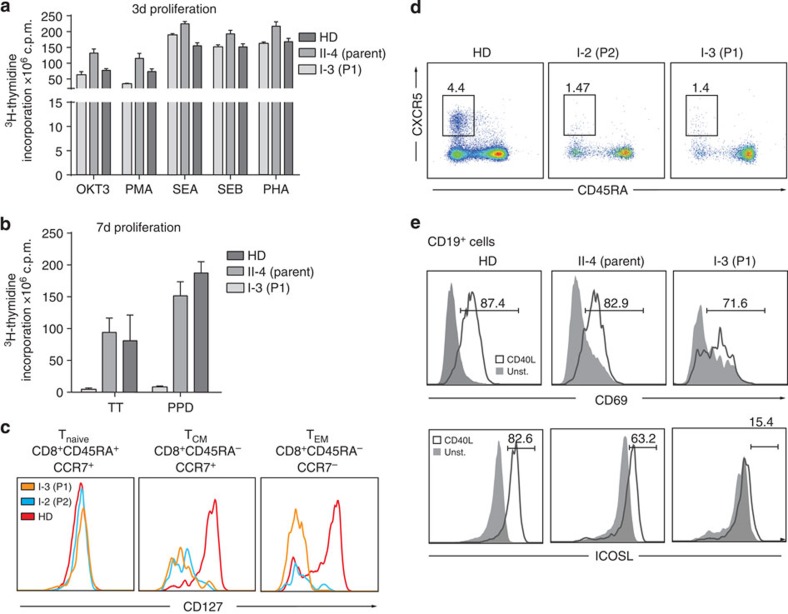
Effects of NIK^Pro565Arg^ on T cells. (**a**) Proliferative response of T cells determined by [^3^H]-thymidine incorporation assay after stimulation with various stimuli after 3 days (OKT3, anti-CD3 antibody (clone OKT3); PMA, phorbol 12-myristate 13-acetate; SEA, Staphylococcal enterotoxin A; SEB, Staphylococcal enterotoxin B; PHA, phytohaemagglutinin) and (**b**) after 7 days stimulation with specific antigens tetanus toxoid (TT) and purified protein derivatives of *M. tuberculosis* (PPD). Mean fold enrichment from one experiment is shown. Error bars denote ±s.d. from three technical replicates. (**c**) Flow cytometric analysis of the CD127 expression in healthy donor (red), P1 (orange) and P2 (blue) in T_naive_ (CD8^+^CD45RA^+^CCR7^+^), T_CM_ (CD8^+^CD45RA^−^CCR7^+^) and T_EM_ (CD8^+^CD45RA^−^CCR7^−^) cell populations. (**d**) Flow cytometric analysis of the T_FH_ cell subset in patient and age matched (9 years) healthy donor. T_FH_ cells defined as CD4^+^CXCR5^+^CD45RA^−^. (**d**,**e**) Plots represent one experiment. (**e**) Flow cytometry analysis of CD69 and ICOSL upregulation on CD40 stimulation on peripheral B cells. PBMCs from healthy donor, parent or P1 were kept unstimulated (grey) or *in vitro* stimulated for 36 h with CD40L (black line). Plots are representative of two independent experiments.

Next, we assessed the effect of NIK^Pro565Arg^ on T cells. Overall CD3^+^CD4^+^ helper T-cell and CD3^+^CD8^+^ cytotoxic T-cell subset distribution was unaffected (Supplementary Fig. 6a), T-cell receptor Vβ repertoires were polyclonal (Supplementary Fig. 7) and regulatory T cells were unaltered (Supplementary Fig. 6b). Upon stimulation of PBMCs with T-cell proliferation stimuli such as anti-CD3 antibody (clone OKT3), phorbol 12-myristate 13-acetate, Staphylococcal enterotoxin A, Staphylococcal enterotoxin B or phytohaemagglutinin, normal proliferative responses were observed (Fig. 6a). In contrast, when the antigen-specific stimuli tetanus toxoid or purified protein derivatives of *Mycobacterium tuberculosis* were used, proliferative responses were severely reduced (Fig. 6b) despite prior tetanus and Bacillus Calmette–Guérin vaccination. These observations prompted us to assess the presence of naive and memory T-cell subsets in both patients. The relative proportions of CD4^+^ effector memory T cells (T_EM_) (markers CD4^+^CD45RA^−^CCR7^−^ or CD4^+^CD45RA^−^CD27^−^, respectively) were comparable between patients and healthy controls and were within the normal age-matched range^32^ (Supplementary Fig. 6c,d and Supplementary Table 3). Relative numbers of CD8^+^ memory T cells from both patients were also within the highly variable normal range^32^. However, P1 exhibited a remarkable expansion of CD8^+^ T_EM_ cells and terminally differentiated effector memory T cells (T_EMRA_, identified as CD8^+^CD45RA^+^CD27^−^ or CD8^+^CD45RA^+^CCR7^−^; Supplementary Fig. 6c,d), possibly attributable to the persistent cytomegalovirus (CMV) viremia in P1 (Supplementary Table 2). Increased IL7R/CD127 expression on CD8^+^ T cells identifies long-lived memory cells^33^,^34^. Similar to Nik-deficient mice^35^, we found dramatically reduced CD127 expression on CD8^+^ memory T cells, on CD8^+^ T_CM_ and on CD8^+^ T_EM_ in both P1 and P2 (Fig. 6c), pointing towards impaired memory responses to viral infections observed in P1 (Fig. 6b and Supplementary Table 2).

Interaction of inducible co-receptor ICOS with ICOS ligand (ICOSL) is important for the differentiation of follicular helper T_FH_ cells and for memory responses of both T and B cells^36^. T_FH_ cells localize to GC reactions within secondary lymphoid organs where they interact with B cells to aid antibody production and maturation^37^. T_FH_ cell numbers are reduced in ICOS-deficient common variable immunodeficiency and in CD40L or CD40 deficiency^37^. Indeed, we found decreased proportions of T_FH_ cells (identified as CXCR5^+^CD45RA^−^) in both patients compared with an age-matched healthy donor (Fig. 6d) and with previously reported healthy donors^38^. As T_FH_ cell development is dependent on ICOSL expression on B cells which is controlled by non-canonical NF-κB signalling^39^, we hypothesized that NIK^Pro565Arg^ causes reduced ICOSL expression leading to impaired T_FH_ generation. Thus, we stimulated PBMCs with CD40L for 36 h and monitored ICOSL expression by flow cytometry. Patient, control parent and healthy donor B cells responded to CD40L stimulation by inducing CD69 expression; however, although control cells were able to upregulate ICOSL, the patient cells failed to do so (Fig. 6e). In summary, NIK^Pro565Arg^ patients exhibit defective differentiation into T_FH_ and impaired function of memory T-cell subsets.

### Decreased numbers and functional impairment of NK cells

**Figure 7:**
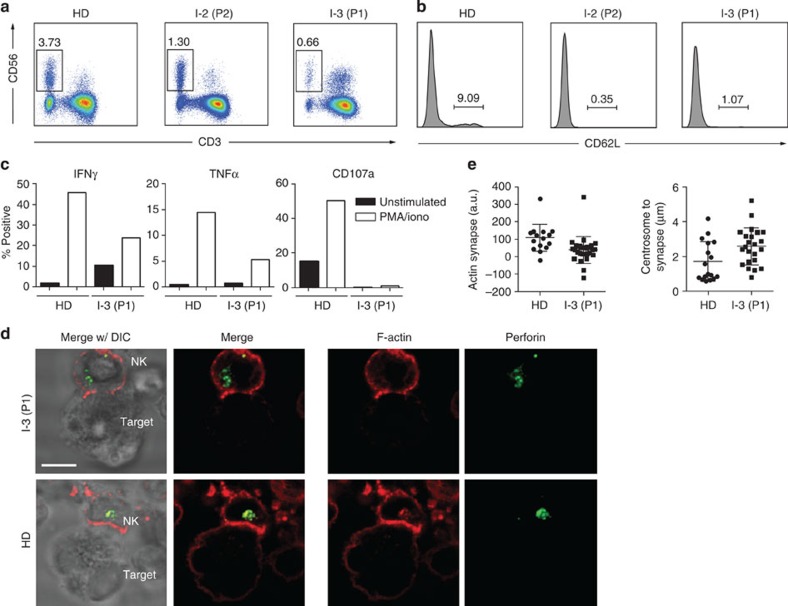
Functional impairment of NIK-deficient NK cells. (**a**) Flow cytometric analysis of NK cells in patients and healthy donor. NK cells defined as CD56^+^CD3^−^. (**b**) Histogram of expression of CD62L on NK cells of patients and healthy donor (gated on CD56^+^CD3^−^ cells). (**c**) Functional response to PMA and ionomycin of NK cells from NIK-deficient patients and healthy donor. PBMCs from healthy donor (HD, left) or patient 1 (P1, right) were incubated with vehicle control (black) or PMA and ionomycin (white) then fixed, permeabilized and analysed by flow cytometry for intracellular expression of IFNγ, TNFα and CD107a. (**a**–**c**) Data represent one experiment. (**d**) Immunofluorescence analysis of mature immunological synapse formation by NK cells. Immunofluorescence detection of perforin (green) and F-Actin by phalloidin (red) in an NK-cell conjugate from Patient 1 (P1, top panel) or healthy donor (HD, bottom panel). K562 cells were used as target cells. DIC, differential interference contrast. White bar indicates 5 μm. Image is representative of one experiment. (**e**) Quantification of immunological synapse formation. *n*=16 (healthy donor) and *n*=27 (P1) for actin synapse quantification, *n*=17 (healthy donor) and *n*=23 (P1) for centrosome to synapse distance quantification; mean ±s.d. shown. Differences are significant as determined by student’s two-sided *t*-test (*P*=0.0073 and *P*=0.0190, respectively).

Consistently low NK-cell numbers in both patients (Supplementary Table 3) along with the susceptibility to CMV, for which NK-cell-mediated defense is relevant, prompted a detailed phenotypic analysis of NK cells. Both patients bearing the NIK^Pro565Arg^ mutant had low numbers of NK cells in the peripheral blood, particularly P1 (Fig. 7a). Despite their low frequency, NK cells showed normal expression of the cell-surface markers perforin, CD16, CD69, CD57 and NKG2C, hallmarks of acquisition of cytotoxic function (Supplementary Fig. 8). Both CD56^bright^ and CD56^dim^ NK cells, representing subsequent stages of NK cell development, were present (Fig. 7a and Supplementary Fig. 8). In addition, patients expressed markers associated with pre-terminal NK-cell developmental stages, including CD117, CD27, CD11a, KIR2DL4 and CD94, at levels comparable to healthy donors (Supplementary Fig. 8). The notable exception was CD62L, which was expressed by a markedly lower proportion of NK cells with NIK^Pro565Arg^ (Fig. 7b).

To determine whether the NK cells present were able to exert cytolytic function and cytokine secretion, we performed activation with phorbol 12-myristate 13-acetate and ionomycin. Notably, production of interferon-γ and TNFα were markedly reduced in patient NK cells, in comparison with stimulated healthy donor cells (Fig. 7c). Although patient NK cells expressed comparable levels of perforin compared with healthy donor cells (Supplementary Fig. 8), they degranulated at a significantly lower frequency as measured by the expression of CD107a (LAMP1) on the cell surface after stimulation (Fig. 7c).

To further define the cytolytic potential of these cells, we evaluated key components of cytotoxicity by quantitative confocal microscopy. Patient-derived NK cells failed to accumulate F-actin at the immunological synapse following incubation with susceptible targets (Fig. 7d,e). In addition, lytic granules failed to polarize to the lytic synapse (Fig. 7e). In concert with the flow cytometric analyses, these data suggest a marked inability of NIK^Pro565Arg^ NK cells to become activated and exert cytolytic function.

## Discussion

TNFα receptor family signalling is essential for B-cell immunity in humans as illustrated by deficiencies in CD40L, CD40 and BAFFR^40^. NIK is an integral component of the non-canonical NF-κB pathway downstream of these receptors^5^.

Studies in the mouse *aly* mutant^8^,^9^ and *Nik* knockout mice^3^ described B-cell deficiency due to disorganized lymph nodes, Peyer’s patches and splenic architecture, accompanied by B-cell lymphopenia and low serum Ig levels due to compromised CSR and SHM^41^. We here identify patients with biallelic mutation in NIK, leading to loss-of-function of the kinase function of NIK. We show that human functional NIK deficiency recapitulates phenotypes described in the mouse studies including B-cell lymphopenia, impaired CSR and SHM, decreased marginal zone and memory B cells, and hypogammaglobulinemia. Although ethic considerations prevented us from obtaining patient biopsies to further investigate secondary lymphoid organ structures, the absence of lymph nodes on repeated clinical examinations suggests that secondary lymphoid organs may be disturbed on loss-of-function of NIK, similar to the observations in mouse models^3^,^8^,^41^.

To extend the murine studies on NIK function in B cells, we investigated the survival properties of patient peripheral blood B cells. *In vitro* stimulation of BAFFR together with BCR and TLR9 resulted in absence of B cells, suggesting a profound survival defect. This is supported by earlier studies showing that Nik overexpression or expression of the NikΔT3 mutant in mice (resistant to Traf3-mediated degradation) leads to increased survival of B cells^42^. Here we find significantly decreased expression of the anti-apoptotic gene *BCL2* in peripheral NIK^Pro565Arg^ B cells, leading to reduced survival. This is most probably the result of impaired BAFF signalling, as NIK is an integral molecule downstream of BAFFR required for B-cell survival^2^,^29^,^30^. Recently, a common variant in *BAFFR* has been shown to modulate NF-κB signalling albeit without effects on survival and subset composition of B cells^43^. Although we identified this variant in our patients, its presence could not explain the B-cell defects described in this study. BAFFR signalling also activates the expression of the B-cell maturation marker CD21 on transitional B cells^2^,^30^,^44^. CD21-deficient mice display severely impaired GC B-cell development and T-cell-dependent B-cell responses^45^ due to reduced GC B-cell survival^46^. CD21 deficiency in humans leads to reduced class-switched memory B cells and hypogammaglobulinemia^47^. Therefore, the reduced CD21 expression on peripheral patient B cells we observed may provide an additional explanation for B-cell survival defects in NIK^Pro565Arg^ patients.

Stimulation with CD40L and IL4, a cytokine with potent anti-apoptotic activity mediated by Stat6-dependent upregulation of Bcl-xL^26^, led to CSR with nearly normal frequency, but delayed kinetics, indicating that at least a proportion of patient B cells was responsive to these stimuli, and that the CSR process itself is largely functional. As CD40 stimulation can signal both via canonical and non-canonical NF-κB pathways leading to AID expression and Igγ germline transcription^48^, our findings indicate that CD40L-mediated, NIK-independent NF-κB signalling contributes to CSR. Delayed CSR kinetics might also be explained by an increased proportion of transitional B cells in patient PBMCs, which reacted more slowly to the B-cell activation stimuli, similar to BAFF-deficient B cells^2^.

As the clinical phentoype suggested a combined immunodeficiency and because recent studies have focused on the role of NIK in T cells^35^,^49^,^50^, we aimed at investigating T-cell functions and T-cell interplay with B cells in patients carrying NIK^Pro565Arg^. Previous studies indicate that NIK-dependent NF-κB signalling is required for ICOSL expression on activated B cells, directing T_FH_ differentiation via interaction with ICOS^39^. T_FH_ cells in turn stimulate B-cell differentiation by expressing CD40 and IL21 (ref. 37). This intimate cell–cell communication leads to the formation of GCs, structures essential for generation of high-affinity antibody responses. Abrogated ICOSL upregulation on B cells and reduced T_FH_ cells in the here described patients probably contribute to impaired GC formation; however, *in situ* analysis of GCs were precluded by ethical constraints.

Another lymphocyte communication process dependent on CD40 signalling via CD4^+^ T-cell help is CD8^+^ T-cell memory maintenance. Subsequent stimulation of IL7R expression is characteristic and essential for CD8^+^ memory T-cell survival^4^. Therefore, lack of IL7R expression on both Nik-deficient mouse^35^ and human (this study) CD8^+^ memory T cells may contribute to the inability of T cells with NIK^Pro565Arg^ to respond to tetanus toxoid and tuberculin despite prior vaccination. Recently, data from ICOSL-deficient patients has implicated ICOSL costimulation in maintenance of memory populations^51^, possibly providing an additional explanation for memory defects in patients carrying NIK^Pro565Arg^.

Patients exhibiting defects in canonical NF-κB signalling such as NEMO^52^,^53^ or IKKβ deficiency^54^ and IκBα hypermorphism^55^ also show antibody deficiencies. Consistent with multiple roles of canonical NF-κB signalling, they show pleiotropic defects throughout the adaptive and innate immune system and developmental defects. Patients with heterozygous mutations in *NFKB2/p100* have recently been described with B-cell deficiency and autoimmunity^56^. Although showing similar manifestations, functional NIK deficiency is more severe than the heterozygous *NFKB2* mutation in humans (this study) and mice^5^. This may be due to the increased p100 levels in NIK^Pro565Arg^ B-LCL that we detected, potentially caused by compensatory increase of NIK-independent canonical NF-κB signalling, which can upregulate p100 expression^19^. In line with this, unprocessed p100 is known to specifically sequester and inhibit RelB^5^, leading to a severe signalling defect.

CD40-dependent IL12 secretion by monocytes is crucial in the defense against mycobacteria. The disseminated Bacillus Calmette–Guérin infection observed in P1 illustrates mycobacterial susceptibility similar to NEMO^57^ and IKKβ-deficient patients^54^. As NIK can signal via both non-canonical and canonical pathway, NF-κB response to mycobacteria may depend on NIK, suggesting that functional NIK deficiency causes defective CD40 signalling in monocytes as well.

Recently, impaired NK-cell function has been recognized for several primary immunodeficiencies (reviewed in ref. 58) most often manifesting in susceptibility to infection by herpesviruses (for example, CMV). NK-cells were persistently decreased in patients carrying NIK^Pro565Arg^. Interestingly, CD62L expression, a marker linked to mature NK-cell subsets with stronger cytolytic functions^59^, was downregulated on patient NK cells. Indeed, although they acquired appropriate developmental and maturity markers, including perforin, patient cells failed to become activated as shown by impaired cytokine secretion, degranulation and polarization of lytic granules towards targets. NIK has not been linked to NK-cell function so far. As canonical NF-κB signalling has been implicated in NK-cell activation^60^, these signals may depend on NIK and therefore mirror NK-cell phenotypes such as those found in CD40L or NEMO deficiencies.

Interestingly, despite the potential defects in secondary lymphoid organ organization discussed above, allogeneic haematopoietic stem cell transplantation (aHSCT) improved the overall condition of patient P1, suggesting that functional NIK deficiency is amenable to aHSCT to at least partially correct the disease. The fatal outcome for P2 (who had received aHSCT without prior conditioning and succumbed shortly after a second aHSCT including conditioning) suggests that at least reduced-intensity conditioning is indicated for successful treatment using aHSCT.

In sum, we identify functional NIK deficiency as a novel, pervasive combined primary immunodeficiency syndrome. Our data revealed an unexpectedly broad range of phenotypic aberrations affecting B-, T- and NK-lineages, and thus highlight essential roles for NIK and adequate control of non-canonical NF-κB signalling for generation and maintenance of the human immune system.

## Methods

### Patient and ethics

This study has been approved by the ethics committee at the Medical University of Vienna, Austria. Biological material was obtained on informed consent in accordance with the Declaration of Helsinki. Clinical data from the patients was provided in anonymized form by the responsible physician(s). The patients were evaluated, followed up and treated at the Department of Immunology at Ankara University in Turkey, covered by the local ethics agreement.

### Homozygosity mapping

Affymetrix 6.0 SNP-based homozygosity mapping was performed in both patients and in both parents of P1, to map homozygous intervals common to both patients but not present in the parents.

Ten microlitres of 50 ng μl^−1^ DNA from the patients were used. The protocol was carried out according to the Affymetrix Genome-Wide Human SNP Nsp/Sty 6.0 protocol. The results were analysed using the Affymetrix Genotyping Console software and PLINK whole genome data analysis toolset (http://pngu.mgh.harvard.edu/~purcell/plink/) as previously described^61^.

### Exome sequencing

A multiplexed 50-bp paired-end read ES was carried out for P1 on Illumina HiSeq2000 Sequencer running on HiSeq Control Software 1.4.8, Real Time Analysis Software 1.12.4.2. The sample preparation used 1 μg of genomic DNA fragmented using Illumina TruSeq DNA Sample Preparation Guide and the Illumina TruSeq Exome Enrichment Guide version 3. The DNA fragment clusters generated ran in a multiplexed pool with five other samples distributed on three lanes of the flow cell.

The data analysis was carried out as previously described^62^ using Burrows–Wheeler Aligner to align the reads to the human genome 19. Insertion/deletion realignment was performed as well as GATK (Genome Analysis Toolkit) base quality score recalibration. For SNV and insertion/deletion calling, Unified Genotyper and GATK Variant quality score recalibration was performed. SNV and insertion/deletion lists were uploaded to SeattleSeq Annotation database. Known variants (present in 1000Genomes or dbSNP build 137, date of accession: 2 January 2012) were excluded and the lists were filtered for nonsense, missense and splice-site variants present within the homozygous regions detected in both patients and absent in the parents. In addition, ENSEMBL, UCSC, NCBI and EVS public SNP databases (date of accession: 20 February 2012) were interrogated for presence of the variant.

The validation and segregation of the variants found in the final hit list from ES were performed using capillary sequencing on genomic DNA from both patients and family members as described below.

### Variant validation by capillary sequencing

Primers for the variants detected with whole ES were designed using ExonPrimer software from the Helmholtz Center Munich (http://ihg.gsf.de/ihg/ExonPrimer.html) and PrimerZ^63^, respectively, and ordered from Sigma Aldrich, Austria. PCR amplification of the detected variants was performed using Expand High Fidelity PCR System (Roche, Basel, Switzerland).

Capillary sequencing of amplicons was performed on the Applied Biosystems 3130xl Genetic Analyzer capillary sequencer running 3130xl Genetic Analyzer Data Collection Software v3.0, using Big Dye Terminator v3.1 Cycle Sequencing Kit (Applied Biosystems, Germany). Sequence Analysis Software Version 5.2 was used for analysis of the sequences and heterozygous signals with ambiguity code were indicated when 25% of the signal intensity was exceeded. Reads were aligned to reference sequences using the Sequencher software, version 4.10.1.

### *In silico* analyses and modelling of NIK protein structure

The algorithms SIFT^64^, PolyPhen2 (ref. 65) (website accessed: 27 March 2012) and CUPSAT^66^ (website accessed: 9 December 2013) tools were used to predict the effect of the identified mutation on protein function.

To obtain a hypothesis about the change in protein structure and dynamics of the NIK variant compared with the wild-type, molecular dynamics (MD) simulations of NIK^wild-type^ and NIK^Pro565Arg^ were performed using the coarse-grained model FREADY^67^ implemented in MOIL^68^ molecular modelling package. We initiated the MD simulations from the crystal structure of NIK (PDB ID 4G3D, chain A^14^) and let it run for 50 ns at 300 K. In the MD simulations, residues farther than 9 Å from the mutated residue were fixed to the experimental structure. Protein structures were aligned and visualized using MacPyMol (The PyMOL Molecular Graphics System, Version 1.3 Schrödinger, LLC).

### Flow cytometry-based immunophenotyping and cell sorting

Immunophenotyping characterization was performed on a BD LSR Fortessa, BC FACS Canto or BD FACS Calibur. In brief, PBMCs from the patients, parents and healthy donors were isolated using Ficoll density gradient centrifugation and either stored frozen in liquid nitrogen and thawn at a later time point or immediately stained for 20 min at 4 °C with mouse anti-human antibodies using the following volumes for one million cells in 100 μl: 2 μl CD3-APC-H7 (SK7), 3 μl CD4-APC (RPA-T4), 5 μl CD8-V500 (RPA-T8), 5 μl CD8-APC-H7 (SK1), 3 μl CD21-PE (B-ly4), 10 μl CD25-PE (M-A251), 1 μl CD27-BV421 (M-T271), 10 μl CD27-FITC (L128), 2.5 μl CCR7-PE-CF594 (150503), 3 μl IgD-FITC (IA6-2), 10 μl CD86-FITC (2331, FUN-1), 5 μl CD95-PECy7 (DX2), 20 μl IL21R-PE (17A12) (all from BD Biosciences); 3 μl CD56-PE (N901) (from Beckman-Coulter); 2 μl CD3-BV711 (OKT3), 1.5 μl CD4-BV510 (OKT4), 0.2 μl CD45RA-BV605 (HI100), 5 μl CD127-BV421 (A019D5) (all from BioLegend); 2 μl CD3-APC (SK7), 3 μl CD4-PerCP-Cy5.5 (RPA-T4), 3 μl CD19-PerCPCy5.5 (HIB19), 5 μl CD69-APC (FN50), 3 μl ICOSL-PE (B7-H2; clone MIH12), 2.5 μl Foxp3-FITC (PCH101) (all from e-Bioscience); and 10 μl CXCR5-APC (51505; R&D Systems).

T and NK cells were evaluated using around 1 × 10^6^ PBMCs. The analysis of the B-lymphocyte compartment was performed using around 4 × 10^6^ cells as previously described^2^,^69^,^70^. NK cells were stained as described previously^71^. For intracellular staining, PBMCs were activated for 3 h with 50 mg ml^−1^ ionomycin and 5 mg ml^−1^ phorbol myristate acetate in the presence of Brefeldin A and antibodies to CD107a and cell surface markers^72^. Cells were then fixed and permeabilized with Cytofix/Cytoperm solution (BD Biosciences).

All analyses were performed using FlowJo X (TreeStar Inc.) and data were graphed with Prism 6.0 (GraphPad Software).

Flow cytometry-based sorting of peripheral B-cell populations stained with anti-CD19-PerCP-Cy5.5, anti-CD27-BV421, anti-CD3-APC-H7 and anti-IgD-FITC as described above was performed on ultra-high-speed six-way digital cell sorter from Beckmann Coulter at the Medical University Vienna Flow Cytometry Core Facility.

Magnetic microbeads based sorting of peripheral B cells was performed using anti-CD20 paramagnetic labelling according to the manufacturer’s instructions (130-091-104, Miltenyi Biotec, Bergisch-Gladbach, Germany).

### Quantitative real-time PCR analysis

Extraction of RNA from sorted B cells was performed using RNeasy kit from Qiagen, first-strand complementary DNA synthesis was done using Expand Reverse Transcriptase from Roche using both oligo-dT and random hexamer primers, and gene expression was analysed by quantitative PCR using Kappa Sybr Fast qPCR MasterMix ABI Bioprism from Kappa Biosystems on 7900HT Fast Real-Time PCR System from Applied Biosciences according to manufacturers’ instructions.

Intron-spanning primers were used for the gene expression analysis. The primer sequences are as follows: *BCL2*-forward 5′- CCGGGAGATGTCGCCCCTGGTGGA -3′, *BCL2*-reverse 5′- AGGCCGCATGCTGGGGCCGTA -3′; *MCL1*-forward 5′- TCGTAAGGACAAAACGGGAC -3′, *MCL1*-reverse 5′- ACCAGCTCCTACTCCAGCAA -3′; *BCL2L1*-forward 5′- GAATGACCACCTAGAGCCTTGG -3′, *BCL2L1*-reverse 5′- TGTTCCCATAGAGTTCCACAAAAG -3′; *GAPDH*-forward 5′- TGATGGCATGGACTGTGGTC -3′, *GAPDH*-reverse 5′- TTCACCACCATGGAGAAGGC -3′.

### Cell culture and stimulation conditions

Healthy donor, patient and family members PBMCs (isolation as above) and Epstein–Barr virus transformed B-cell line were maintained in RPMI-1640 medium supplemented with 10% of inactivated FCS (Life Technologies, Gibco), 50 U ml^−1^ penicillin, 50 μg ml^−1^ streptomycin and 292 μg ml^−1^ L-glutamin (all from Gibco) at 37 °C in a humidified atmosphere with 5% CO_2_. PBMC stimulation conditions and reagents were trimeric human CD40L and human BAFF-Fc, both produced as described^2^, and human recombinant IL21 (ebiosciences) used at 20 ng ml^−1^; B-LCL stimulation conditions were hBAFF (R&D Systems; 2149-BF-010) used at 50 ng ml^−1^ for 6–12 h; and primary fibroblast stimulation conditions were Lymphotoxin α1/β2 (R&D Systems, 678-LY-010) used at 50 and 100 ng ml^−1^ from 15 min to 4 h and TNFα (14-8329-62; eBioscience) used at 20 ng ml^−1^ from 15 min to 4 h.

Primary fibroblasts and HEK293 cells were cultured in glucose-rich DMEM (PAA), supplemented and cultured as above.

B-cell activation assays were performed by stimulating PBMCs with CD40L and IL21 as described^2^ or using CD40L and IL4 (100 ng ml^−1^, ImmunoTools) in Iscoves’s modified DMEM medium (Invitrogen), supplemented with 10% heat-inactivated FCS (Biowest), 100 U ml^−1^ penicillin (Invitrogen), 100 μg ml^−1^ streptomycin (Invitrogen), 1 μg ml^−1^ insulin (Sigma-Aldrich), 1 μg ml^−1^ reduced glutathione (Sigma-Aldrich), 2.5 μg ml^−1^ apo-transferrin (Sigma-Aldrich), 2 mM glutamine (Life Technologies, Gibco) and 1% non-essential amino acids (Gibco). Culture was started with equal number of cells and samples were analysed at days 3, 6 and 9 by flow cytometry.

### Immunoblot analysis

Protein was isolated using cell lysis buffer containing 20 mM Tris (pH7.5), 150 mM NaCl, 2 mM EDTA, 1% TritonX-100 (pH7.1) and complete protease inhibitor cocktail (Sigma Aldrich). Polyvinylidene difluoride or nitrocellulose membranes were prepared according to standard methods. Primary antibodies used for immunoblot analysis of NF-κB pathways were: rabbit anti-human IKKα (2682), phospho-IKKα/β (2697), p100/p52 (4882), NIK (4994), RelB (clone C1E4), TRAF3 (4729), p65 (clone D14E12), p105/p50 (3035) and mouse anti-human IκBα (clone L35A5), all purchased from Cell Signaling and used at 1:1,000 dilution. For detection of tagged recombinant proteins, anti-human c-Myc (551101, BD Biosciences) was used at 1:1,000 dilution and horseradish peroxidase-coupled anti-HA (H6533, Sigma-Aldrich) was used at 1:3,000 dilution. For loading controls, mouse anti-human GAPDH (clone 6C5; Santa Cruz Biotechnology) and anti-human RCC1 (clone E-6; Santa Cruz Biotechnology) were used at 1:1,000 dilution. Horseradish peroxidase-conjugated goat anti-rabbit (Bio-rad) and goat anti-mouse (BD Biosciences) secondary antibodies were used at 1:10,000 or 1:50,000 dilution and detected using a chemiluminescent substrate (Amersham ECL Prime Western Blotting Detection Reagent, GE Life Sciences) together with Hyperfilm ECL (Fischer Scientific).

### Kinase assay

HEK293 cells were transfected in six-well plates with NIK- and/or IKKα-tagged expression vectors generated by gateway recombination using the pTO (carboxy-terminal streptavidin–haemagglutinin tag) or pCS2 (amino-terminal 6 × myc tag) destination vectors^73^, lysed 24 h post transfection in lysis buffer containing 20 mM HEPES (ph7.9), 20% glycerol, 50 mM KCl, 400 mM NaCl, 1 mM EDTA, 1 mM dithiothreitol, 5 mM β-glycerophosphate, 1 mM phenylmethyl sulphonyl fluoride, 5 mg ml^−1^ apronitin, 10 mM NaF, 5 mg ml^−1^ leupeptin and 5 mM Na_3_VO_4_, and subsequently subjected to SDS–PAGE and immunoblot analysis.

### Amplification and sequence analysis of *IGH* transcripts

IgA and IgG transcripts were amplified from cDNA of thawed PBMCs using subgroup-specific forward primers in the leader sequence of *IGHV3* and *IGHV4* in combination with a Cα or Cγ consensus reverse primer^22^,^74^. All PCR products were cloned into the pGEM-T easy vector (Promega) and prepared for sequencing on an ABIPRISM 3130xl (Applied Biosystems). Obtained sequences were analysed with the IMGT database (http://www.imgt.org/) for *IGHV*, *IGHD* and *IGHJ* use, and mutation analysis^75^.

### T-cell proliferation analysis

T-cell proliferation assays were carried out as described previously^62^,^76^.

### T-cell CDR3 Vβ spectratyping

TCR Vβ spectratyping was performed as previously described^77^ with minor modifications. The primers used were as before with the following exceptions: Primers for variable regions, BV02-5′- ACATACGAGCAAGGCGTCGA -3′, BV04-5′- CATCAGCCGCCCAAACCTAA -3′, BV07-5′- CAAGTCGCTTCTCACCTGAATGC -3′, BV17-5′- TGTGACATCGGCCCAAAAGAA -3′, BV21-5′- GGAGTAGACTCCACTCTAAG -3′, BV24-5′- CCCAGTTTGGAAAGCCAGTGACCC -3′; primers for constant regions (used for BV05, BV06BC, BV20), CβB1- 5′- CGGGCTGCTCCTTGAGGGGCTGCG -3′; FAM-marked constant primer-5′- ACACAGCGACCTCGGGTGGG -3′.

Sequences were acquired using an ABI 3130xl Sequencer (ABI Applied Biosystems) and analysed using GeneMapper software version 4.0.

### Immunostaining of lymphotoxin-stimulated fibroblasts

Fibroblasts of patient (P2) and healthy donor were stimulated with 100 ng ml^−1^ of lymphotoxin α1/β2 (R&D Systems, 678-LY-010) for 4 h. After stimulation, cells were fixed with 4% formaldehyde in PBS for 30 min and then blocked and permeabilized with solution containing 10% FCS plus 0.1% Triton X-100. Cells were immunostained with DAPI and rabbit antibodies against NFκB2 (p100/p52) (Cell Signaling, 3017) and NF-κB (p105/p50) (Cell Signaling, 3035) at a dilution of 1:100, respectively, and afterwards with anti-rabbit Alexa Fluor 546-conjugated antibody at a dilution of 1:500 (Life Technologies, A10040). Images were acquired on a Leica AF6000 fluorescent microscope using Leica LASAF software for acquisition. Images were taken at × 64 magnification.

### Reconstitution assay

cDNA encoding for wild-type human *MAP3K14* was cloned into a bicistronic retroviral pMMP vector coexpressing *MAP3K14* and enhanced green fluorescent protein (eGFP) marker gene via IRES sequence. RD114-pseudotyped retroviral particles were generated by transfection into HEK293 cells using the calcium chloride transfection method (8 μg retroviral vector DNA, 12 μg gag/pol DNA, 5 μg RD114 DNA) in the presence of 25 μM chloroquin (Sigma-Aldrich, C6628) for 12 h. Supernatants containing viral particles were collected after 24, 36 and 48 h. Viral titration was performed on HT-1080 cells. Patient and normal donors fibroblast cells were transduced with retroviral particles in the presence of 8 μg ml^−1^ polybrene (Santa Cruz, sc-134220) for 12 h. Transduction efficiency was determined by eGFP expression by FACS analysis and was between 45% and 70%. After transduction, immunofluorescence studies were performed as above with additional staining against GFP used at a dilution of 1:100 (antibody sc-69779, Santa Cruz). Slides were visualized as above. Data was graphed using Prism 6.0 (GraphPad Software).

### Confocal microscopy of immunological synapses

Cell conjugates were formed for the evaluation of the immunological synapse by confocal microscopy as previously described^78^. Following fixation and permeabilization, conjugates were incubated with anti-perforin Alexa Fluor 488 (Biolegend) at a dilution of 1:50 and Phalloidin Alexa Fluor 568 at a dilution of 1:100. Images were acquired on a Leica SP8 laser scanning confocal microscope. Excitation was by tunable white light laser and detection of emission by hybrid gallidium (HyD) detectors. Acquisition was controlled by Leica LASAF software and images were subsequently exported to Volocity software (PerkinElmer) for analysis. Data were graphed using Prism 6.0 (GraphPad Software).

## Author contributions

K.L.W., S.K., E.S.-V., W.G., E.M., I.B., E.S., C.D.-C., H.S and P.P.B. performed experiments. F.D., S.H., M.G.B. and A.I. provided clinical care of the patients, provided clinical data and were involved in clinical care including allogeneic HSCT of both patients. A.K. performed HSCT of P1. E.M., P.P.B. and J.S.O. performed NK-cell analyses. M.C.vZ. performed T-cell phenotyping and Ig sequence analysis. P.M. performed computational modelling and *in silico* prediction algorithms. W.F.P. performed T-cell proliferation assays. G.V. assisted with imaging. G.S.-F., A.C.-N. and J.C. were involved in critical scientific discussions. K.B. conceived this study, provided laboratory resources, and together with K.L.W., S.K. and E.S-V. planned, designed and interpreted experiments. K.L.W, S.K., I.B. and K.B. wrote the manuscript with input from H.E., M.C.vZ., J.S.O, G.S.-F., E.M., C.D.-C., E.S.-V. and A.I. All authors critically reviewed the manuscript and agreed to its publication.

## Additional information

**How to cite this article:** Willmann, K. L. *et al*. Biallelic loss-of-function mutation in NIK causes a primary immunodeficiency with multifaceted aberrant lymphoid immunity. *Nat. Commun.* 5:5360 doi: 10.1038/ncomms6360 (2014).

## Supplementary Material

Supplementary Information Supplementary Figures 1-9, Supplementary Tables 1-5, Supplementary Note 1 and Supplementary References
